# Postoperative stereotactic radiosurgery (SRS) vs hypofractionated stereotactic radiotherapy (SRT) for resected brain metastases – a single centre analysis

**DOI:** 10.1007/s10585-025-10334-5

**Published:** 2025-02-10

**Authors:** Lena Kretzschmar, Hubert Gabrys, Anja Joye, Johannes Kraft, Matthias Guckenberger, Nicolaus Andratschke

**Affiliations:** 1 Department of Radiation Oncology, University Hospital Zurich, Zurich, Switzerland; 2 Department of Radiation Oncology, Kantonsspital Aarau, Aarau, Switzerland

**Keywords:** Brain metastases, Postoperative, Radiosurgery, Hypofractionated stereotactic radiotherapy

## Abstract

Postoperative radiotherapy improves local control (LC) after resection of brain metastases. In comparison to whole brain radiotherapy (WBRT) stereotactic radiosurgery (SRS) to resection cavity significantly reduces cognitive side effects. However, two phase-III trials have reported suboptimal LC with SRS, leading to increased interest in hypofractionated stereotactic radiotherapy (SRT) as an alternative to improve outcomes. This single-centre study, based on a prospective quality assurance protocol, included 161 patients with 185 resected brain metastases treated with either SRS or SRT between February 2018 and June 2023. Patients were assigned to treatment based on cavity size, with SRS typically used for cavities < 10 cc and SRT for larger volumes. Primary and secondary endpoints were LC and radiation necrosis (RN), respectively. Data analysis was conducted retrospectively. Median cavity size was 13.3 cc, with 20% of cavities receiving SRS and 80% SRT. 12-month LC was 92.6% (95-CI: 88.2 – 97.3%), 12-month RN incidence was 9% (95-CI: 3–14%), with RN limited to CTCAE v5 ≤ 2. In cavities < 10 cc, no significant difference in LC was found between SRS and SRT. For cavities > 10 cc, PTV volume was the only significant predictor of LC, while fractionation and dose did not significantly impact outcomes. SRS and SRT both offer excellent LC for resection cavities < 10 cc with low rates of RN, suggesting SRS as the preferred treatment in this collective, in consideration of patient comfort and resource allocation. In larger cavities, PTV volume significantly influences LC. Dose escalation might be beneficial in improving outcomes in these cases.

**Supplementary Information:**

The online version contains supplementary material available at 10.1007/s10585-025-10334-5.

## Introduction

Postoperative radiotherapy significantly improves local control rates after resection of single or multiple brain metastases when compared to observation alone [1, 2]. In the past years, the approach of postoperative radiotherapy has shifted from whole brain radiotherapy (WBRT) to single-fraction stereotactic radiosurgery (SRS) of the resection cavity, after radiosurgery was shown to maintain reasonable local control in a phase-III-trial by Mahajan et al. [3], as well as significantly reducing late side effects like cognitive impairment when compared to WBRT, as shown in another landmark trial by Brown et al. [4].

Yet, in both Mahajan et al. [3] and Brown et al. [4], 12-month local control at cavity site proved worse after SRS (61% and 72% respectively), not only when compared to WBRT, but also in comparison to SRS alone in other trials, that showed excellent 12-month local control rates of 80% and more [5, 6]. Most likely the relatively large number of higher-volume cavity sizes treated with lower single-fraction radiosurgery doses due to toxicity concerns are responsible for this observation. Larger cavity sizes and marginal doses < 16 Gy have been identified as independent predictors of inferior local control after postoperative SRS in the past [7]. In this context Mahajan et al. [3] proposed SRS dose-escalation for larger cavity sizes in future trials, suggesting the use of hypofractionated stereotactic radiotherapy (SRT) as an alternative strategy to provide higher biologically effective radiation doses to the PTV. Several retrospective trials had shown highly promising 12-month local control of up to 93% with the use of SRT in larger cavity sites before [8, 9].

In 2020 a systematic review including 3458 patients by Akanda et al. showed a significantly better 12-month local control for cavities treated with SRT in comparison with SRS (LC12 87.3% vs. 80%, *p* = 0.021), while maintaining low toxicity rates [10]. While hypothesising that the use of SRT could be beneficial in the postoperative treatment of brain metastases in general, the authors also expressed an inability to determine the influence of possible confounders in their review due to the heterogeneity of the individual studies. Some studies in the meta-analysis focused specifically on larger cavity sizes, while others did not report on resection cavity size at all, or mixed SRS and SRT approaches.

Altogether, while there are multiple studies reporting the feasibility of single and fractionated stereotactic treatment approaches for resected brain metastases alike, there is still a distinct lack of studies directly comparing the two approaches (SRS vs. SRT) and simultaneously controlling for confounders like dose and cavity size, especially when shifting the focus to smaller resection cavities.

After the publication of the two landmark trials [3, 4] on postoperative radiosurgery for resection cavities, we adapted our treatment strategy for resected brain metastases in our centre from historical SRT to single-fraction SRS in cavities smaller than 10 cc, and collected follow-up data on these patients starting from February 2018. We applied the hypothesis that smaller resection cavities can be safely treated with SRS to maintain high local control, while larger metastases would benefit from the historically implemented SRT according to the aforementioned retrospective data [8, 9].

To our knowledge, this is the first clinical analysis directly comparing individual patient data for local control in SRS vs. SRT for resected brain metastases with a focus on cavity size as a predictor of outcome in terms of local control and toxicity.

## Materials and methods

### Study design and patient selection

This single-centre study is based on a prospective patient assignment and data collection as part of a prospective quality assurance and follow-up program implemented for all patients receiving stereotactic radiotherapy for brain metastases. Therefore, the comparison of SRS versus SRT was not pre-specified and thus the analysis with assessment of local control and radiation necrosis is retrospective in nature. All patients with resected brain metastases receiving postoperative radiotherapy to one or more resection cavities with SRS or SRT in our clinic at University Hospital Zurich between February 2018 and June 2023 were included. We excluded patients who received re-irradiation to the same cavity (defined as Type-1-Re-irradiation according to Andratschke et al. [11]) or whose treatment failed to be completed according to prescription. Patients with previous radiotherapies to other intact or resected brain metastases in the past that showed no geometrical overlap (defined as Type-2-Re-irradiation or Repeat organ irradiation according to Andratschke et al. [11]) were included in the study. There were no restrictions imposed regarding primary tumour entities. Our prospective observation/follow-up period was locked by end of December 2023 to allow for a minimum follow-up of 6 months.

### Treatment and follow-up

Patients were treated with single- (SRS) or multi-fraction stereotactic radiotherapy (SRT). Treatment planning was performed CT- and MRI-based: CT-images were constructed with a slice thickness of 0.6 mm and performed after contrast application unless contraindications for iodine contrast medium were present; for cranial MRI a post-contrast axial T1-sequence (slice thickness 0.6–1.0 mm) was acquired and registered with planning CT for contouring. The tumour cavity was defined as clinical target volume (CTV), while the planning target volume (PTV) was generated by 2 mm three-dimensional margin. We applied conformal treatment planning (VMAT), image-guidance (combined CBCT and stereoscopic kV imaging with ExacTrac, Brainlab AG, Munich), stereotactic patient setup with a customized mask and inhomogeneous dose prescription (prescription to 80% isodose). The two approaches differed only in the application of treatment, either in a single fraction (SRS) or in multiple fractions (SRT).

All treatment decisions in our department were made based on the existing literature and our internal clinic guidelines. Cavities/PTVs > 10 cc were generally treated with SRT in 6 fractions of 5 Gy per fraction at 80% isodose level (IDL), or in some cases in 5 fractions with 6 Gy per fraction at 80% IDL, to a cumulative dose of 30 Gy. Cavities/PTVs < 10 cc received SRS with either 20 Gy (< 4 cc), 18 Gy (4.1–7.9 cc) or 17 Gy (8.0–10.0 cc), at 80% IDL respectively, in line with Brown et al. [4]. In individual cases with concerns about increased risk of toxicity by the treating clinician, i.e. proximity of the PTV to critical organs-at-risk (optic chiasm, brain stem), SRT was used as an alternative to SRS also in smaller PTVs. Additionally, in very large PTVs (> 30 cc) or multiple, grouped cavities with added concern for toxicity, fractionation of SRT was modified to 10 fractions with 3 Gy per fraction at 80% IDL.

Generally, patients were followed up with regular imaging and physical examination every three months for the first year after SRS or SRT, and every six months afterwards or until intracranial progression. Standard image modality for follow-up was brain MRI with standard sequences (T1 +/- gadolinium; T2 / FLAIR). If deemed necessary by the treating primary radiation oncologist, this modality was complemented by Fluoroethyl-L-tyrosine-PET/MRI, for differentiating tumour progression from post therapeutic changes or identifying radiation necrosis.

### Ethical approval

This study was approved by our institutional ethics board as well as the state ethics committee of Switzerland (BASEC ID 2018 − 01794).

### Data analysis

All demographic, clinical and imaging data were recorded and filed in Microsoft Excel (version 16.0).

R (version 4.3.0; R Foundation for Statistical Computing, Vienna, Austria) and RStudio (version 2023.06.0; Posit, PBC, Boston, MA, USA) were used for statistical analysis, and modelling solutions are based on Harrison et al. in “R for Health Data Science” [12].

Descriptive statistics (median, range) were used to describe patient and treatment baseline characteristics. Overall survival (OS) and freedom from intracranial progression, as well as local recurrence at cavity site and incidence of radiation necrosis were calculated from the end of treatment course, with time-to-event curves being created using the Kaplan-Meier method and cumulative incidence calculations. Univariate analysis (t-testing) was applied to analyse PTV volume in relation to local recurrence and incidence of radiation necrosis. Multivariate Cox proportional hazards regression was used to determine the impact of different factors (PTV volume, number of fractions, dose per fraction and total dose) over the entire patient collective and in the subgroup of cavity sizes < 10 cc. For the multivariate analyses one variable was selected and included per 10 events each. In general, the threshold for statistical significance was set at *p* ≤ 0.05.

**Fig. 1:**
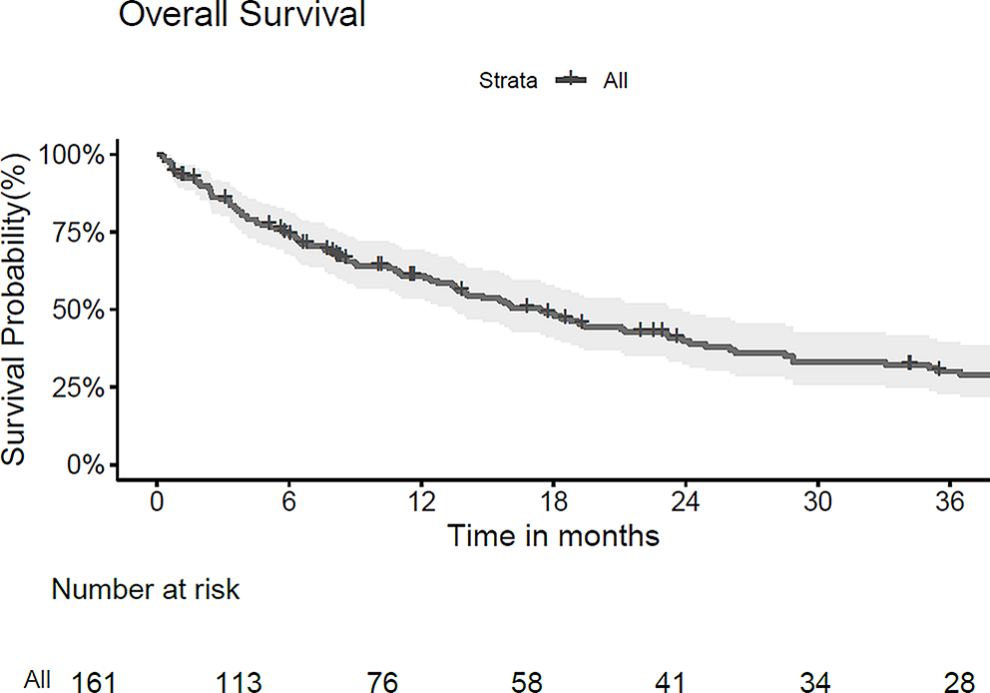
Kaplan-Meier-curve for overall survival

## Results

A total of 161 patients with 185 resected and irradiated metastases were included in the analysis. Most patients had a lung (*n* = 64, 40%) or melanoma (*n* = 53, 33%) primary. 80% of cavities (*n* = 148) received fractionated, stereotactic radiosurgery (SRT), single-fraction stereotactic surgery (SRS) was applied to 20% (*n* = 37) of cavities (Tables 1 and 2). Cavity sizes ranged from 0.89 cc to 98.9 cc, with a median of 13.3 cc. Median overall survival was 17.4 months (95% CI 13.5–23.9 months; Fig. 1), median time to intracranial progression was 8.8 months (95% CI 6.8–14.8 months). 9.7% (*n* = 18, crude estimate) local recurrences were observed during follow-up period until December 2023. Only one of these local recurrences developed in isolation, all others were accompanied by distant brain failure, most often (in all but one case) synchronous with local failure. However, isolated distant brain failure without local recurrence remained the most common pattern of intracranial recurrence (in *n* = 68 of 85 patients with intracranial recurrence in total, crude estimate). A total of 26 patients developed leptomeningeal recurrence on MRI during follow-up, equaling 16% (crude estimate) of all patients who received postoperative SRS/SRT – 4 of these patients showed simultaneous local recurrence at cavity site. In terms of toxicity, radiation-induced necrosis was observed in 15 resected and irradiated metastases (8.1%) and limited to CTCAE v5 grade 1 and 2 (asymptomatic or only corticosteroids indicated); no CTCAE v5 grade ≥ 3 radiation necroses were observed among the patient collective.

**Table 1:**
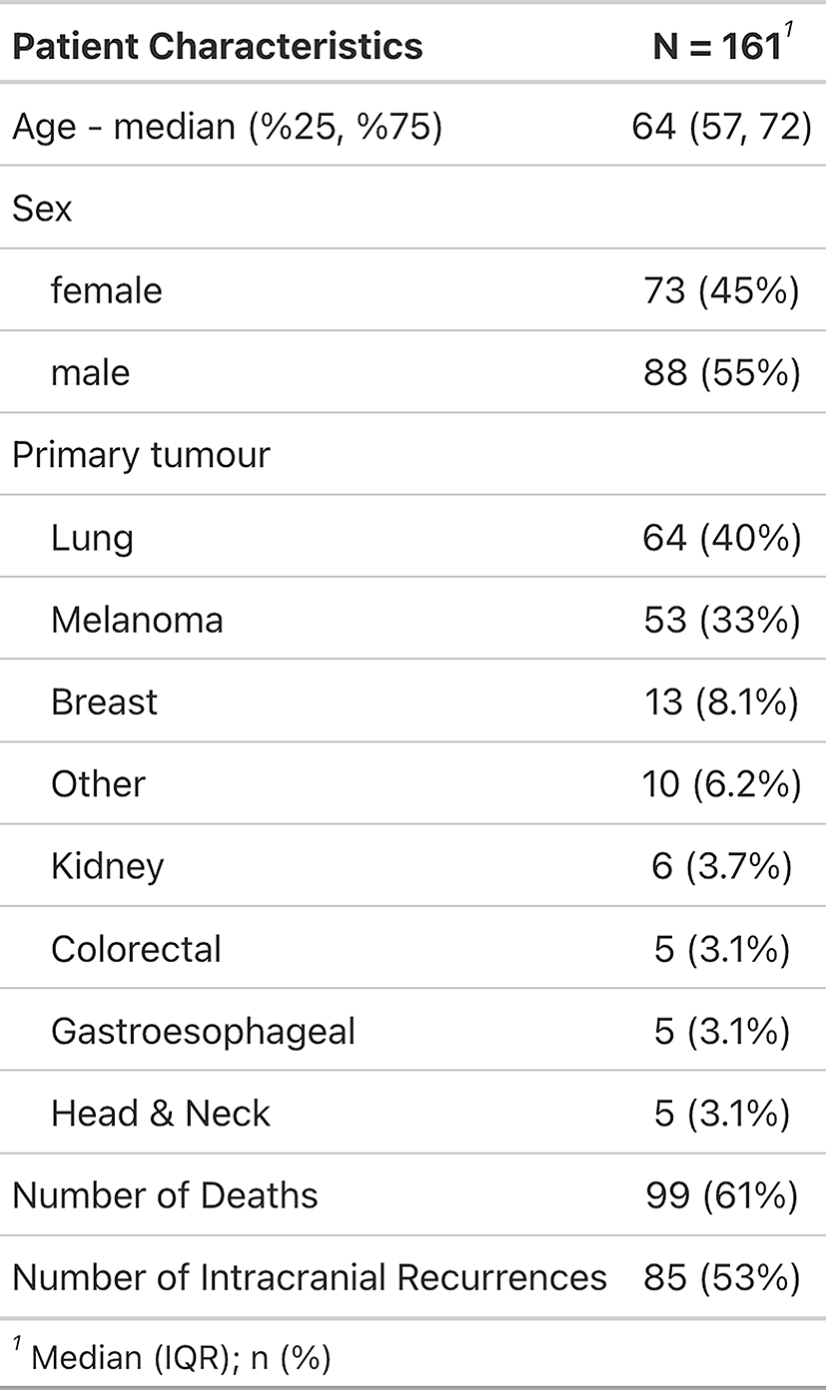
Baseline patient characteristics

**Table 2:**
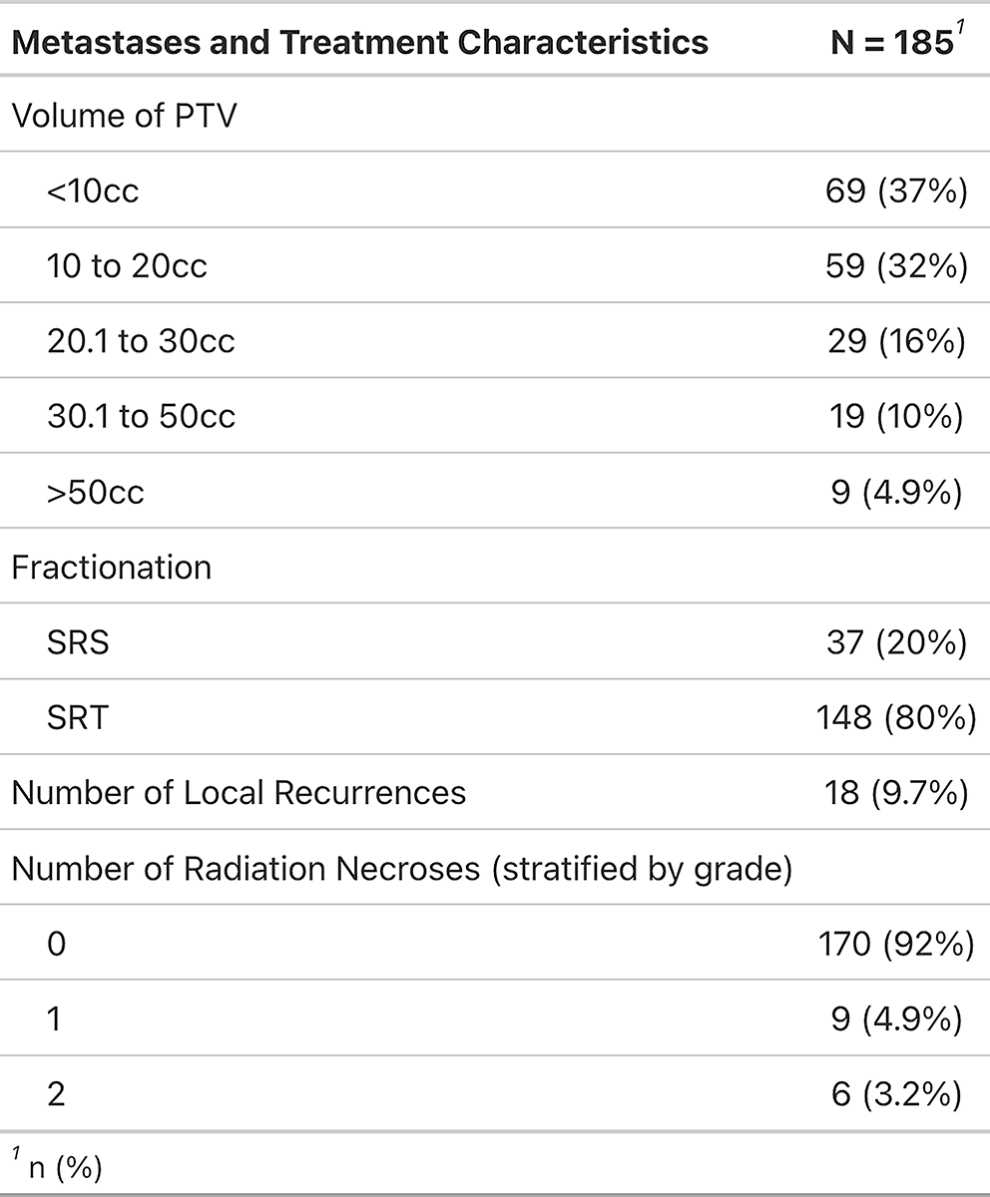
Metastases and treatment characteristics

**Fig. 2:**
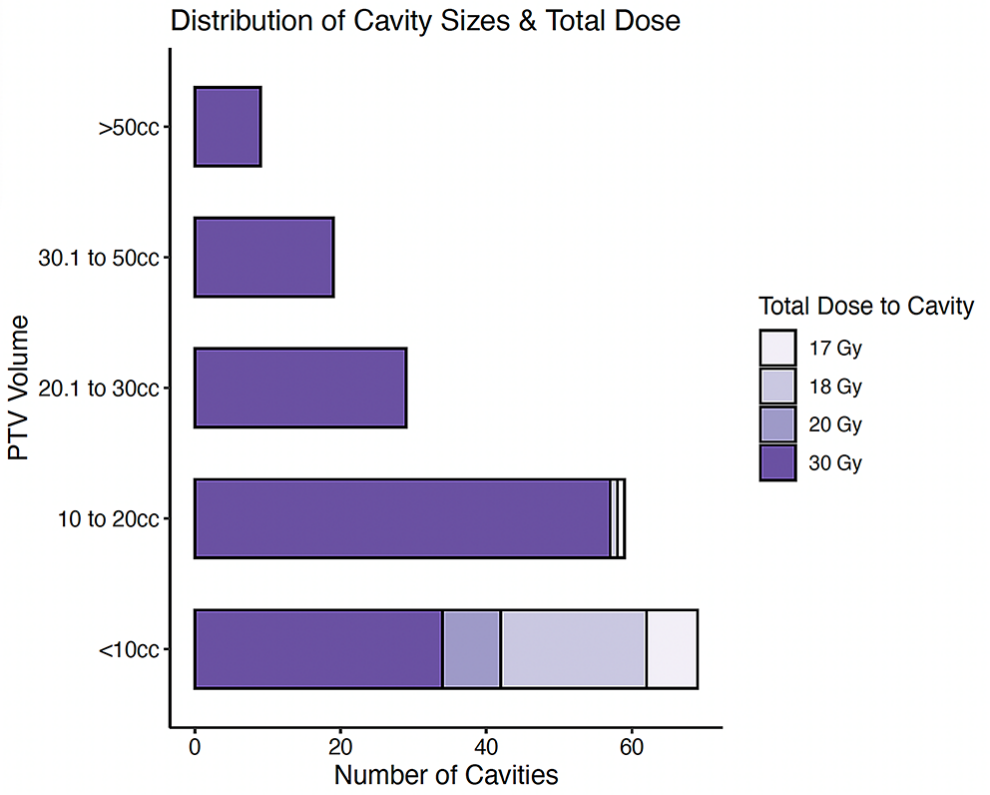
Distribution of cavity sizes and total dose

As seen in Fig. 2a significant number of cavities (*n* = 34) received fractionated stereotactic radiotherapy (SRT) up to a total dose of 30 Gy, despite being smaller than 10 cc and therefore formally amendable for single-fraction stereotactic radiosurgery (SRS) according to our institute’s standard of care. These deviations were made at the discretion of the treating clinician, and mostly due to concerns of toxicity in cases of i.e. irradiation of multiple clustered cavities at the same time or proximity to organs-at-risk.

Cumulative incidence calculations and Cox proportional hazard regressions showed a statistically significant impact on the likelihood of local recurrence at cavity site only for the volume of the irradiated PTV in cc (HR 1.02, *p* = 0.016), but not for fractionation (SRS vs. SRT), primary tumour entity or total dose.

**Fig. 3:**
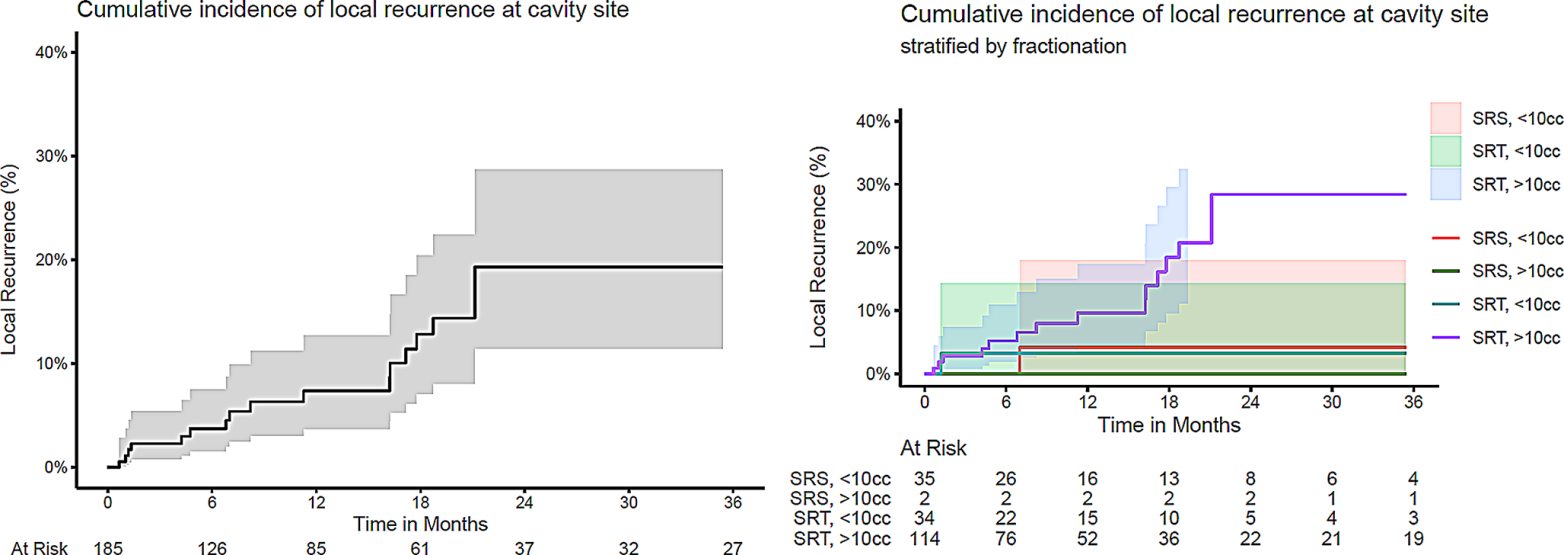
Cumulative incidences of local recurrence at cavity site (All / Stratified by fractionation & PTV-volume)

The relatively widespread use of SRT for cavities < 10 cc in the patient collective enabled us to perform a subgroup analysis of all cavities treated with SRS (*n* = 37) and those smaller than 10 cc treated with SRT (*n* = 34) to directly compare local control rates between them, as seen further below (Fig. 3; Table 3). In contrast, two cavities were treated with SRS despite being > 10 cc in volume. The exception was made due to both volumes being close in size to 10 cc (10.2 cc and 12.8 cc respectively), distant from any organs at risk, and the patient-individual wish to be treated in a single fraction due to claustrophobia.In the subgroup of patients who received SRS or SRT for small cavities < 10 cc, none of the analysed variables (volume of PTV in cc, fractionation or total dose) had a statistically significant effect on the likelihood of local recurrence. In the subgroup of larger cavities > 10 cc only volume of PTV had a statistically significant effect (Fig. 3; Table 3).

**Table 3:**
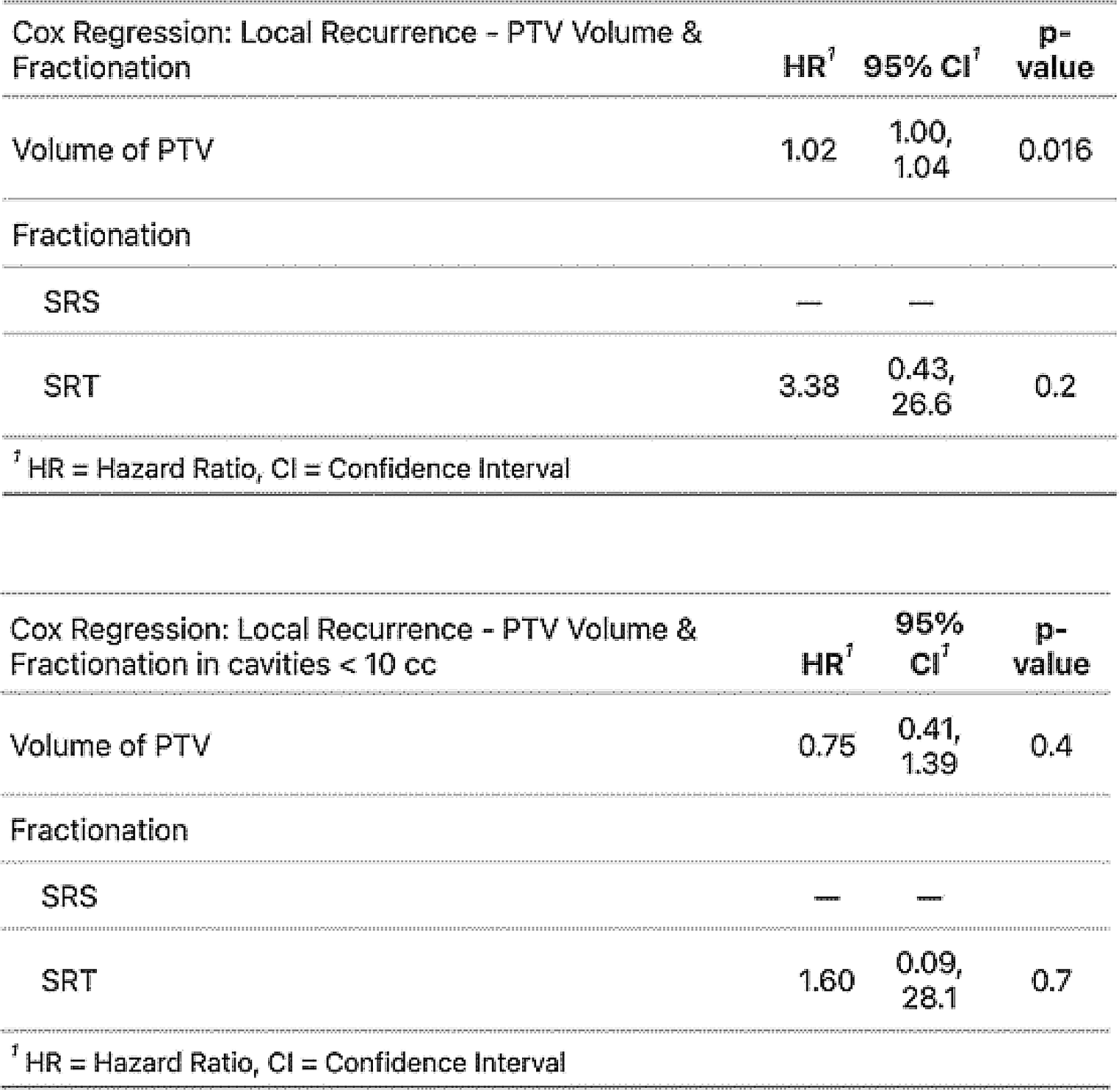
Cox-proportional hazard regressions (local recurrence – PTV volume in cc & fractionation) in patient collective and subgroup < 10 cc

**Fig. 4:**
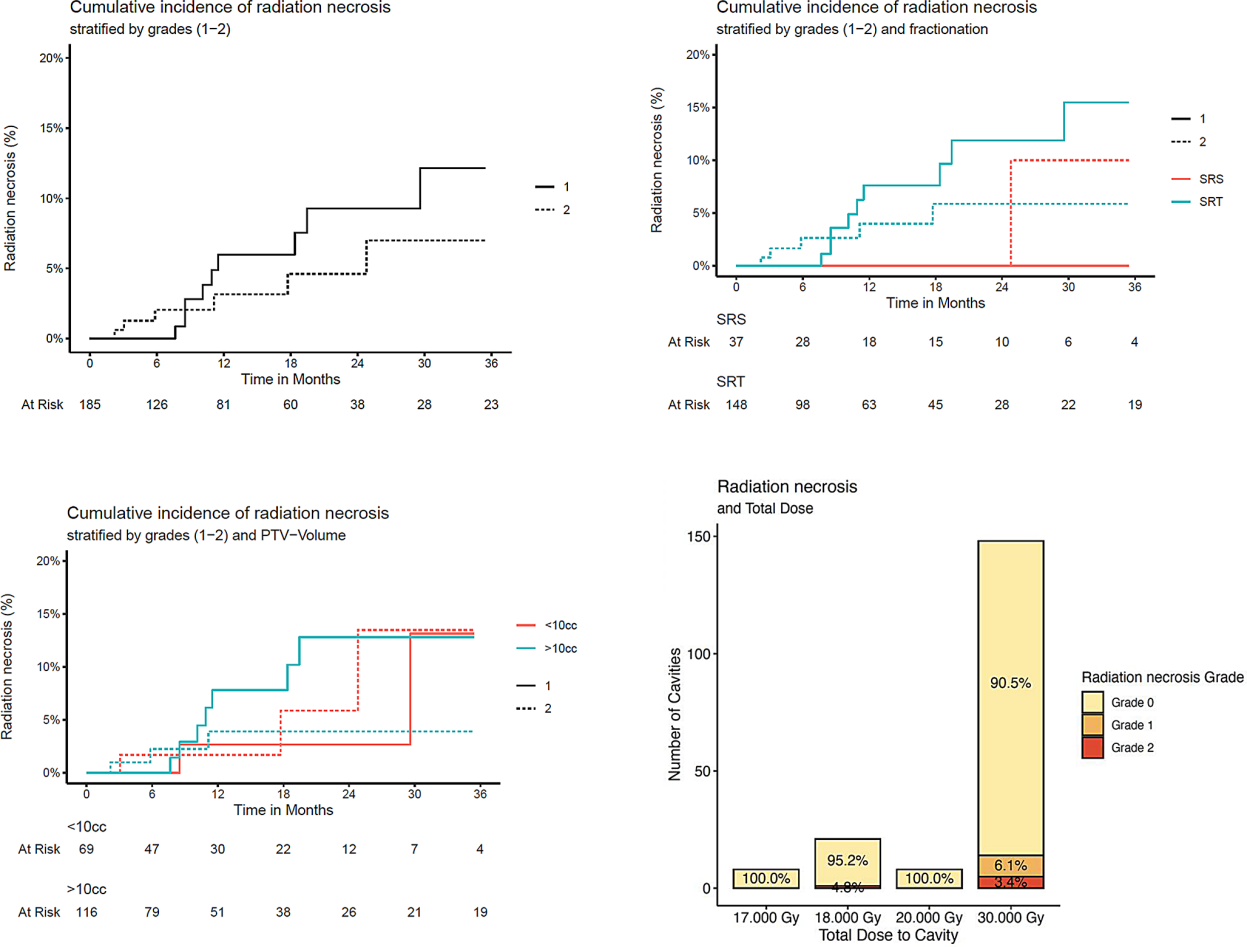
Cumulative incidences of radiation necrosis at cavity site (All, Stratified by fractionation, Stratified by PTV-volume) and overview with total dose

The likelihood of developing radiation necrosis was not significantly influenced by either PTV-volume (cc), fractionation (SRS vs. SRT), primary tumour or total dose (Fig. 4; Table 4).

**Table 4:**
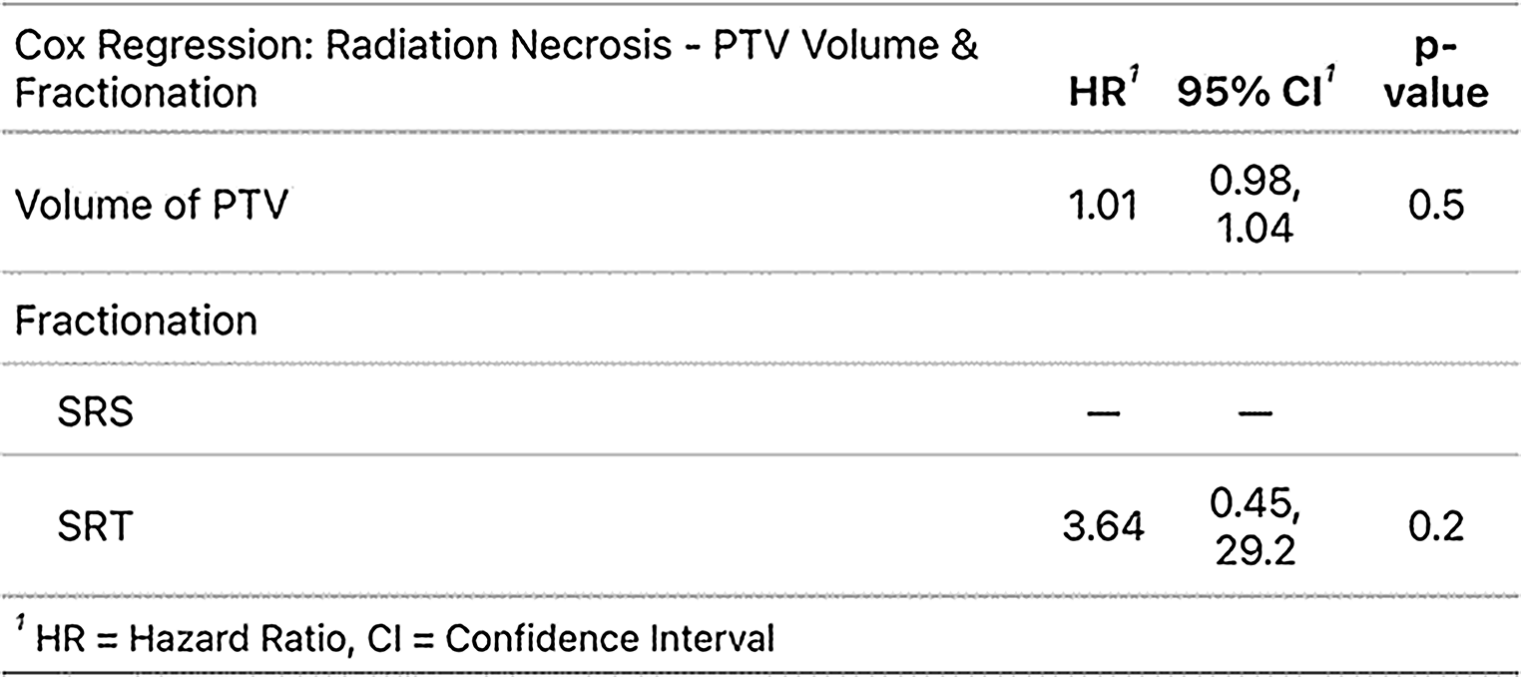
Cox-proportional hazard regression (Radiation necrosis)

A detailed compilation of the performed multivariate analyses can be found in the Appendix.

## Discussion

Historically, hypofractionated stereotactic radiotherapy (SRT) has been frequently employed instead of whole brain radiotherapy for postoperative treatment of the resection cavity, although prospective evidence for this approach is still missing. Recently, single-fraction radiosurgery (SRS) after resection of brain metastases has been investigated in two landmark trials by Mahajan et al. [3] and Brown et al. [4], showing that it is effective in terms of local tumour control and associated with reasonable toxicity rates, making it the de facto standard for postoperative radiotherapy. Nevertheless, several retrospective studies and systematic reviews [8–10] have indicated that SRT might lead to better local control rates when compared to SRS, while maintaining low toxicity, especially in the management of large cavities. However, the question of whether SRT is generally superior to SRS in terms of local control remains unanswered due to limited and scattered evidence.

Currently, two phase-III-trials – by Brown et al. [13] and by Waltenberger et al. [14] – are under way, searching to directly compare the two approaches of post-operative SRS and SRT with a primary endpoint of local failure in a controlled, prospective setting. First preliminary data from these trials is expected in 2025 with mature results evolving later. Until then, more readily available data is needed to guide treatment approaches.

In our analysis of a total of 185 resection cavities in 161 patients treated with SRS or SRT between February 2018 and June 2023 according to the internal guidelines of our clinic, we observed overall excellent local control of 92.6% (95-CI: 88.2 – 97.3%) and 80.7% (95-CI: 72.4 − 90.0%) after 12 and 24 months, respectively. These results compare favourably with the existing literature, with Akanda et al. reporting local control rates of 83.7% at 12 months in their meta-analysis [10]. For the subgroup of cavities > 10 cc in our study, the absolute majority (*n* = 146 of 148) of which were treated with SRT with a total dose of 30 Gy, local control remained good (1-year-LC of 90.6%, 95-CI: 84.4 − 97.2%, and 2-year-LC of 72.7%, 95-CI: 61.4 − 86.1%, respectively). This falls in line with the results from a recent analysis by Lehrer et al. that specifically included brain metastases postoperatively treated with hypofractionated, stereotactic radiotherapy [15], summarising 12-month local control rates at around 90%.

In comparison, the two landmark, randomised phase-III-trials by Mahajan et al. [3] and Brown et al. [4] reported much lower 12-month local control rates of 61% and 72% respectively. In Brown et al. [4] doses of 20 Gy were used if the cavity volume was less than 4·2 cc, 18 Gy if 4·2–7·9 cc, 17 Gy if 8·0–14·3 cc, 15 Gy if 14·4–19·9 cc, 14 Gy if 20·0–29·9 cc, and 12 Gy if 30·0 cc or more up to the maximal surgical cavity extent of 5 cm. Mahajan et al. [3] utilized similar prescription doses of 16 Gy (for ≤ 10 cc), 14 Gy (for 10·1–15 cc), and 12 Gy (for > 15 cc). Akanda et al. [10] reported a median dose of < 18 Gy with a 12 month local control rate of 80.0% (ranging from 76 to 84%) in 14 of the studies that were included in their meta-analysis. 8 studies (9 data sets) employed a median dose of ≥ 18 Gy with a 12 month local control rate of 81% (ranging from 78 to 85%), and 2 studies did not state their median dose. A more in-depth analysis of the irradiation schemes (i.e. a report of median dose of all fractionated schemes allowing for further comparison between them, or a conversion into BED or EQD2 doses) was not included in the scope of the systematic review. Although median dose did not significantly influence local control, which might be partially owed to the lack of more intensified subgroup analysis, multi-fraction, stereotactic radiotherapy showed a statistically significant, positive impact on local control at 12 months in comparison to single-fraction, stereotactic radiotherapy [10].

Another meta-analysis by Lehrer et al. [16], that investigated single vs. multi-fraction stereotactic radiotherapy in large brain metastases (> 14 cc), also showed numerically inferior local control at 12 months for patients receiving SRS (random effects estimate: 68%) when compared to those receiving SRT (random effects estimate: 86.8%) in the postoperative subgroup. A comparison of these two groups did *not* yield statistical significance, however, possibly due to the span of reported local control at 12 months between the included studies, ranging from 34 up to 100%, leading – in turn – to a large overlap in the 95% confidence intervals.

In our study we did not see a statistically significant difference between SRS or SRT in local control rates for cavities < 10 cc. Cavities > 10 cc which were treated with SRT showed a volume-dependent correlation with local failure. The size of the resection cavity has been shown to be an independent predictor for worse local control [8]. Possible reasons for this have been extensively discussed in the literature: Firstly, the surgical cavity maximal diameter correlates with the initial maximal tumor diameter, reflecting a larger and, possibly, deeper microscopic invasion front at the margins [17]. Secondly, due to lower oxygenation of tumour beds compared to intact brain metastases, higher radiation doses for comparable local control might be required – a problem exacerbated in larger cavities with more hypoxic tissue [18]. In single-fraction settings, underdosing larger target volumes due to toxicity concerns is common – this, too, has been described as an independent predictor for inferior local control [7].

The results of our study show that SRS is equivalent to SRT in the treatment of cavities < 10 cc with comparable local control and toxicity rates, being the preferred treatment modality in this collective due to its advantages in patient comfort and cost effectiveness. Despite the SRT approach we have taken for cavities > 10 cc, larger PTV size is still significantly associated with lower local control rates in our study, leading us to hypothesise that further dose escalation in larger cavities might be explored for superior local tumour control. This could be more safely achieved with an SRT approach, with data suggesting rates of symptomatic toxicity, i.e. radiation necrosis grade 3 or greater, are lower compared to a SRS approach in larger cavities [19, 20].

In our cohort, the incidence of radiation necrosis was low (9% at 12 months after SRS/SRT, 95-CI: 3–14%) and no CTCAE v5 grade 3 or higher radiation necrosis occurred. There was also no statistically significant difference between groups if stratified for PTV volume, fractionation (SRS/SRT) or total dose, suggesting room for dose escalation. If, additionally, a deeper microscopic tumour invasion in large resection cavities is assumed, enlarging CTV-to-PTV margins might be another option to improve local control – however, some published data suggests higher rates of radiation necrosis with margin expansion [21].

In the meta-analysis by Lehrer et al. [16] that specifically included larger cavities treated with SRS/SRT (> 14 cc) incidence rates of radiation necrosis ranging from 0 to 38.5% were described, though no statistically significant differences between the applied treatment techniques or doses were observed. The meta-analysis by Akanda et al. [10] describes radiation necrosis rates of < 10% up to 28%, foregoing a group-specific stratification between cavities treated with SRS and SRT due to altogether inconsistent reporting on radiation necroses in almost all of the included trials. Lehrer et al. [16] also discussed the difficulty in reporting accurate rates of radiation necrosis in their meta-analysis due to definition and categorisation varying strongly from centre to centre. This uncertainty on true incidence rates of radiation necrosis illustrates the continued challenge in the diagnosis and reporting of this highly relevant protracted side effect of SRS/SRT, especially in the follow-up of asymptomatic patients, when confined to non-invasive methods, due to uncertainties in the presentation of these lesions on MR-imaging [22]. A certain range of imprecise toxicity estimates is therefore to be expected when investigating incidence of radiation necrosis during follow-up, though specialised imaging modalities like FET-PET have shown to be effective in aiding differential diagnosis [23].

Distant intracranial failure at 12 months was 58% in our study (95-CI: 49 − 66%), and 64% at 24 months after SRS/SRT (95-CI: 54 − 72%) and is in line with existing data: Jensen et al. report 69% distant brain failure within 12 months after SRS to cavity site [24], while Akanda et al. found slightly lower rates of 47.3% in their systematic review [10]. MR-assessed leptomeningeal disease recurrence at 12 months after SRS/SRT was moderate with 23% (95-CI: 14 − 31%), which corresponds with the data published by Mahajan et al. in 2017 [3], but is more than the 12.6% systematically reported by Akanda et al. [10]. These variations in the reported incidence of leptomeningeal spread after SRS/SRT have been taken note of in the literature – with reasons for the inconsistencies being ascribed mostly to diagnostic variability between clinical, MR- and cerebrospinal-fluid-based assessment of leptomeningeal disease as well as discordance in physicians’ assessments thereof [20].

Certain limitations should be kept in mind when interpreting the results of this study: Although it was implemented as part of a prospective quality assurance follow-up, it is retrospective in nature and bound to the established standard of care in a single department. Physician- and patient-specific particularities must be acknowledged as confounders in cases where deviations from our clinic-internal standards of care occurred: Two cavities were treated with SRS despite being slightly > 10 cc in volume due to patient-specific features (wish for single-fraction treatment due to claustrophobia), whereas several (*n* = 34) cavities were treated with SRT, despite being < 10 cc, at the discretion of the treating physician due to toxicity concerns, i.e. irradiation of multiple clustered cavities at the same time/proximity to organs-at-risk. Lastly, with absolute numbers of local recurrences and radiation necroses both being low in our patient collective, statistical analysis of competing risks for these events is, by nature, challenging, giving rise to uncertainties in the results presented.

## Conclusion

In the postoperative treatment of brain metastases with resection cavities < 10 cc, SRS and SRT showed equally excellent local control rates with simultaneously similarly low rates of radiation necrosis, indicating that SRS may be preferred to minimise patient efforts and radiotherapy resources allocation. In cavities > 10 cc volume of PTV postoperative SRT showed adequate local control rates in this subgroup when compared with existing literature, while PTV volume remained an independent predictor for worse local control. The incidence of radiation necrosis was not significantly impacted by volume of PTV, fractionation, or total dose, suggesting room for dose escalation in larger resection cavities as a possible strategy for improving local control rates.

## Electronic supplementary material

Below is the link to the electronic supplementary material.

Supplementary Material 1

## Data Availability

The data that support the findings of this study are not openly available due to reasons of sensitivity and are available from the corresponding author upon reasonable request. Data are located in controlled access data storage at University Hospital Zurich.

## References

[1] Patchell RA, Tibbs PA, Regine WF et al (1998) Postoperative radiotherapy in the treatment of single metastases to the brain: a randomized trial. JAMA 280(17):1485–1489. 10.1001/jama.280.17.1485 9809728 10.1001/jama.280.17.1485

[2] Kocher M, Soffietti R, Abacioglu U et al (2011) Adjuvant whole-brain radiotherapy versus observation after radiosurgery or surgical resection of one to three cerebral metastases: results of the EORTC 22952-26001 study. J Clin Oncol 29(2):131–141. 10.1200/JCO.2010.30.1655 10.1200/JCO.2010.30.1655 PMC3058272 21041710

[3] Mahajan A, Ahmed S, McAleer MF et al (2017) Post-operative stereotactic radiosurgery versus observation for completely resected brain metastases: a single-centre, randomised, controlled, phase 3 trial. Lancet Oncol 18(8):1040–1048. 10.1016/S1470-2045(17)30414-X 10.1016/S1470-2045(17)30414-X PMC5560102 28687375

[4] Brown PD, Ballman KV, Cerhan JH et al (2017) Postoperative stereotactic radiosurgery compared with whole brain radiotherapy for resected metastatic brain disease (NCCTG N107C/CEC-3): a multicenter, randomized, controlled, phase 3 trial. Lancet Oncol 18(8):1049–1060. 10.1016/S1470-2045(17)30441-2 28687377 10.1016/S1470-2045(17)30441-2 PMC5568757

[5] Jensen CA, Chan MD, McCoy TP et al (2011) Cavity-directed radiosurgery as adjuvant therapy after resection of a brain metastasis: clinical article. J Neurosurg 114(6):1585–1591. 10.3171/2010.11.JNS10939 21166567 10.3171/2010.11.JNS10939 PMC3789371

[6] Quigley M, Fuhrer R, Karlovits S et al (2008) Single session stereotactic radiosurgery boost to the post-operative site in lieu of whole brain radiation in metastatic brain disease. J Neurooncol 87:327–332. 10.1007/s11060-007-9515-z 18183353 10.1007/s11060-007-9515-z

[7] Luther N, Kondziolka D, Kano H et al (2013) Predicting tumor control after resection bed radiosurgery of brain metastases. Neurosurgery 73(6):1001–1006. 10.1227/NEU.0000000000000148 24264235 10.1227/NEU.0000000000000148

[8] Minniti G, Esposito V, Clarke E et al (2013) Multidose stereotactic radiosurgery (9 gy x 3) of the postoperative resection cavity for treatment of large brain metastases. Int J Radiation Oncology-Biology-Physics 86(4):623–629. 10.1016/j.ijrobp.2013.03.037 10.1016/j.ijrobp.2013.03.037 23683828

[9] Wang C, Floyd SR, Chang C et al (2012) Cyberknife hypofractionated stereotactic radiosurgery (HSRS) of resection cavity after excision of large cerebral metastasis: efficacy and safety of an 800 cGy x 3 daily fractions regimen. J Neurooncol 106:601–610. 10.1007/s11060-011-0697-z 21879395 10.1007/s11060-011-0697-z

[10] Akanda ZZ, Hong W, Nahavandi S et al (2020) Post-operative stereotactic radiosurgery following excision of brain metastases: a systematic review and meta-analysis. Radiother Oncol 142:27–35. 10.1016/j.radonc.2019.08.024 31563407 10.1016/j.radonc.2019.08.024

[11] Andratschke N, Willmann J, Appelt AL et al (2022) European Society for Radiotherapy and Oncology and European Organisation for Research and Treatment of Cancer consensus on re-irradiation: definition, reporting, and clinical decision making. Lancet Oncol 23(10):e469–e478. 10.1016/S1470-2045(22)00447-8 36174633 10.1016/S1470-2045(22)00447-8

[12] Harrison E, Pius R R for Health Data Science. https://argoshare.is.ed.ac.uk/healthyr_book/. Accessed 31-08-2024

[13] Brown PD et al Single Fraction Stereotactic Radiosurgery Compared With Fractionated Stereotactic Radiosurgery in Treating Patients With Resected Metastatic Brain Disease. Ongoing Trial. https://www.clinicaltrials.gov/study/NCT04114981. Accessed 31-08-2024

[14] Waltenberger M, Bernhardt D, Diehl C et al (2023) Hypofractionated stereotactic radiotherapy (HFSRT) versus single fraction stereotactic radiosurgery (SRS) to the resection cavity of brain metastases after surgical resection (SATURNUS): study protocol for a randomized phase III trial. BMC Cancer 23(1):709. 10.1186/s12885-023-11202-9 37516835 10.1186/s12885-023-11202-9 PMC10385881

[15] Lehrer EJ, Breen WG, Singh R et al (2024) Hypofractionated stereotactic radiosurgery in the management of Brain metastases. Neurosurgery. 10.1227/neu.0000000000002897 38511946 10.1227/neu.0000000000002897

[16] Lehrer EJ, Peterson JL, Zaorsky NG et al (2019) Single versus Multifraction Stereotactic Radiosurgery for large brain metastases: an International Meta-analysis of 24 trials. Int J Radiation Oncol Biology Phys 103(3):618–630. 10.1016/j.ijrobp.2018.10.038 10.1016/j.ijrobp.2018.10.038 30395902

[17] Keller A, Doré M, Cebula H et al (2017) Hypofractionated Stereotactic Radiation Therapy to the Resection Bed for Intracranial metastases. Int J Radiat Oncol Biol Phys 99(5):1179–1189. 10.1016/j.ijrobp.2017.08.014 28974415 10.1016/j.ijrobp.2017.08.014

[18] Prabhu R, Hui-Kuo S, Hadjipanayis C (2012) Current dosing paradigm for stereotactic radiosurgery alone after Surgical Resection of Brain metastases needs to be optimized for Improved Local Control. Int J Radiat Oncol Biol Phys 83(1):e61–e66. 10.1016/j.ijrobp.2011.12.017 22516387 10.1016/j.ijrobp.2011.12.017

[19] Eaton BR, La Riviere MJ, Kim S et al (2015) Hypofractionated radiosurgery has a better safety profile than single fraction radiosurgery for large resected brain metastases. J Neuro-Oncology 123:103–111. 10.1007/s11060-015-1767-4 10.1007/s11060-015-1767-4 25862006

[20] Minniti G, Niyazi M, Andratschke N et al (2021) Current status and recent advances in resection cavity irradiation of brain metastases. Radiat Oncol 16(73). 10.1186/s13014-021-01802-9 10.1186/s13014-021-01802-9 PMC8051036 33858474

[21] Jhaveri J, Chowdhary M, Zhang X et al (2018) Does size matter? Investigating the optimal planning target volume margin for postoperative stereotactic radiosurgery to resected brain metastases. J Neurosurg 130(3):797–803. 10.3171/2017.9.JNS171735 10.3171/2017.9.JNS171735 PMC6195865 29676690

[22] Chao ST, Ahluwalia MS, Barnett GH et al (2013) Challenges with the diagnosis and treatment of Cerebral Radiation Necrosis. Int J Radiat Oncol Biol Phys 87(3):449–457. 10.1016/j.ijrobp.2013.05.015 23790775 10.1016/j.ijrobp.2013.05.015

[23] Schlürmann T, Waschulzik B, Combs S et al (2023) Utility of amino acid PET in the Differential diagnosis of recurrent brain metastases and treatment-related changes: a Meta-analysis. J Nuclear Med 64(5):816–882. 10.2967/jnumed.122.264803 10.2967/jnumed.122.264803 36460343

[24] Jensen CA, Chan MD, McCoy TP et al (2009) Adjuvant radiosurgery after resection of a Brain Metastasis allows for the Delay or Elimination of whole brain Radiotherapy. Int J Radiat Oncol Biol Phys 75(3):S226. 10.1016/j.ijrobp.2009.07.521

