# Supplementary material for: Postoperative stereotactic radiosurgery (SRS) vs hypofractionated stereotactic radiotherapy (SRT) for resected brain metastases – a single centre analysis
Source: Clin Exp Metastasis. 2025 Feb 10;42(2):16. doi: 10.1007/s10585-025-10334-5 (PMC11811445; doi:10.1007/s10585-025-10334-5)
Supplement: Supplementary file 1 — Supplementary Material 1 [file 10585_2025_10334_MOESM1_ESM.pdf]

Clinical & Experimental Metastasis

Kretzschmar Lena[1], Gabrys Hubert[2], Joye Anja[3], Kraft Johannes[4], Guckenberger Matthias[5], Andratschke Nicolaus[6]

"Postoperative stereotactic radiosurgery (SRS) vs hypofractionated stereotactic radiotherapy (SRT) for resected brain metastases – a single centre analysis"

1: Department of Radiation Oncology, University Hospital Zurich, Zurich, Switzerland. **Mail:** Lena.Kretzschmar@usz.ch
2: Department of Radiation Oncology, University Hospital Zurich, Zurich, Switzerland. **Mail:** Hubert.Gabrys@usz.ch
3: Department of Radiation Oncology, University Hospital Zurich, Zurich, Switzerland. **Mail:** Anja.Joye@usz.ch
4: Department of Radiation Oncology, Kantonsspital Aarau, Aarau, Switzerland. **Mail:** johannes.kraft@ksa.ch
5: Department of Radiation Oncology, University Hospital Zurich, Zurich, Switzerland. **Mail:** Matthias.Guckenberger@usz.ch
6: Department of Radiation Oncology, University Hospital Zurich, Zurich, Switzerland. **Mail:** Nicolaus.Andratschke@usz.ch

Appendix: Multivariate Analyses

Cox-Regression 1: Local Recurrence – PTV Volume (continuous) & Primary Tumour.

Cox Regression: Local Recurrence - PTV Volume & Primary	**HR**[1]	**95% CI**[1]	**p-value**
Volume of PTV	1.03	1.01, 1.05	0.005
Primary			
Breast	—	—	
Colorectal	0.00	0.00, Inf	>0.9
Gastroesophageal	0.00	0.00, Inf	>0.9
Head & Neck	0.00	0.00, Inf	>0.9
Kidney	0.00	0.00, Inf	>0.9
Lung	1.04	0.12, 8.76	>0.9
Melanoma	1.38	0.17, 11.0	0.8
Other	0.61	0.03, 11.0	0.7

[1] HR = Hazard Ratio, CI = Confidence Interval

Cox-Regression 2: Local Recurrence – PTV Volume (continuous) & Dose.

Cox Regression: Local Recurrence - PTV Volume & Dose	**HR**[1]	**95% CI**[1]	**p-value**
Volume of PTV	1.02	1.00, 1.04	0.015
Total Dose	1.11	0.93, 1.32	0.3

[1] HR = Hazard Ratio, CI = Confidence Interval

Cox-Regression 3: Local Recurrence – PTV Volume (continuous) & Fractionation.

Cox Regression: Local Recurrence - PTV Volume & Fractionation	**HR**[1]	**95% CI**[1]	**p-value**
Volume of PTV	1.02	1.00, 1.04	0.016
Fractionation			
SRS	—	—	
SRT	3.38	0.43, 26.6	0.2

[1] HR = Hazard Ratio, CI = Confidence Interval

Cox-Regression 4: Local Recurrence – PTV Volume (BINARY, >10cc / <10cc) & Fractionation.

Cox Regression: Local Recurrence - PTV Volume & Fractionation	**HR**[1]	**95% CI**[1]	**p-value**
Volume of PTV (binary)			
<10cc	—	—	
>10cc	3.41	0.64, 18.0	0.15
Fractionation			
SRS	—	—	
SRT	2.21	0.22, 21.7	0.5

[1] HR = Hazard Ratio, CI = Confidence Interval

Cox-Regression 5: Local Recurrence – PTV Volume (continuous) & Fractionation in cavities < 10cc.

Cox Regression: Local Recurrence - PTV Volume & Fractionation in cavities < 10 cc	**HR**[1]	**95% CI**[1]	**p-value**
Volume of PTV	0.75	0.41, 1.39	0.4
Fractionation			
SRS	—	—	
SRT	1.60	0.09, 28.1	0.7

[1] HR = Hazard Ratio, CI = Confidence Interval

Cox-Regression 6: Local Recurrence – PTV Volume (continuous) & Dose in cavities < 10cc.

Cox Regression: Local Recurrence - PTV Volume & Dose in cavities < 10 cc	**HR**[1]	**95% CI**[1]	**p-value**
Volume of PTV	0.77	0.42, 1.39	0.4
Total Dose	1.03	0.80, 1.32	0.8

[1] HR = Hazard Ratio, CI = Confidence Interval

Cox-Regression 7: Radiation Necrosis – PTV Volume (continuous) & Fractionation.

Cox Regression: Radiation Necrosis - PTV Volume & Fractionation	**HR**[1]	**95% CI**[1]	**p-value**
Volume of PTV	1.01	0.98, 1.04	0.5
Fractionation			
SRS	—	—	
SRT	3.64	0.45, 29.2	0.2

[1] HR = Hazard Ratio, CI = Confidence Interval

Cox-Regression 8: Radiation Necrosis – PTV Volume (BINARY, >10cc / <10cc) & Fractionation.

Cox Regression: Radiation Necrosis - PTV Volume & Fractionation	**HR**[1]	**95% CI**[1]	**p-value**
Volume of PTV (binary)			
<10cc	—	—	
>10cc	0.60	0.19, 1.87	0.4
Fractionation			
SRS	—	—	
SRT	6.00	0.69, 51.9	0.10

[1] HR = Hazard Ratio, CI = Confidence Interval

Cox-Regression 9: Local Recurrence – PTV Volume (continuous) & Primary Tumour in cavities > 10cc.

Cox Regression: Local Recurrence - PTV Volume & Primary in cavities > 10 cc	**HR**[1]	**95% CI**[1]	**p-value**
Vol_PTV	1.02	1.00, 1.05	0.043
Primary_factor			
Breast	—	—	
Colorectal	0.00	0.00, Inf	>0.9
Gastroesophageal	0.00	0.00, Inf	>0.9
Head & Neck	0.00	0.00, Inf	>0.9
Kidney	0.00	0.00, Inf	>0.9
Lung	0.80	0.09, 7.18	0.8
Melanoma	1.49	0.19, 12.0	0.7
Other	0.61	0.03, 11.3	0.7

[1] HR = Hazard Ratio, CI = Confidence Interval

Cox-Regression 10: Radiation Necrosis – PTV Volume (continuous) & Primary Tumour in cavities >10cc.

Cox Regression: Radiation Necrosis - PTV Volume & Primary in cavities > 10 cc	**HR**[1]	**95% CI**[1]	**p-value**
Vol_PTV	1.01	0.99, 1.04	0.3
Primary_factor			
Breast	—	—	
Colorectal	0.96	0.00, Inf	>0.9
Gastroesophageal	1.04	0.00, Inf	>0.9
Head & Neck	0.96	0.00, Inf	>0.9
Kidney	0.99	0.00, Inf	>0.9
Lung	65,702,771	0.00, Inf	>0.9
Melanoma	107,826,226	0.00, Inf	>0.9
Other	70,362,297	0.00, Inf	>0.9

[1] HR = Hazard Ratio, CI = Confidence Interval
